# Amyloid-ß-directed immunotherapy for Alzheimer's disease

**DOI:** 10.1111/joim.12168

**Published:** 2014-03-08

**Authors:** L Lannfelt, N R Relkin, E R Siemers

**Affiliations:** 1 Department of Public Health/Geriatrics, Uppsala UniversityUppsala, Sweden; 2 Weill Cornell Medical CollegeNew York, NY, USA; 3 Eli Lilly and Co.Indianapolis, IN, USA

**Keywords:** Alzheimer's disease, amyloid-beta, clinical trials, immunotherapy

## Abstract

Lannfelt L, Relkin NR, Siemers ER (Uppsala University, Uppsala, Sweden; Weill Cornell Medical College, New York, NY; and Eli Lilly and Co., Indianapolis, IN, USA). Amyloid-ß-directed immunotherapy for Alzheimer’s disease. (Key Symposium). *J Intern Med* 2014; **275**: 284–295.

Current treatment options for Alzheimer's disease (AD) are limited to medications that reduce dementia symptoms. Given the rapidly ageing populations in most areas of the world, new therapeutic interventions for AD are urgently needed. In recent years, a number of drug candidates targeting the amyloid-ß (Aß) peptide have advanced into clinical trials; however, most have failed because of safety issues or lack of efficacy. The Aß peptide is central to the pathogenesis, and immunotherapy against Aß has attracted considerable interest. It offers the possibility to reach the target with highly specific drugs. Active immunization and passive immunization have been the most widely studied approaches to immunotherapy of AD. A favourable aspect of active immunization is the capacity for a small number of vaccinations to generate a prolonged antibody response. A potential disadvantage is the variability in the antibody response across patients. The potential advantages of passive immunotherapy include the reproducible delivery of a known amount of therapeutic antibodies to the patient and rapid clearance of those antibodies if side effects develop. A disadvantage is the requirement for repeated infusions of antibodies over time. After more than a decade of research, anti-amyloid immunotherapy remains one of the most promising emerging strategies for developing disease-modifying treatments for AD. In this review, we examine the presently ongoing Aß-directed immunotherapies that have passed clinical development Phase IIa.

## Introduction

Current treatment options for Alzheimer's disease (AD) are limited to medications that reduce dementia symptoms but do not arrest or reverse the underlying neurodegenerative disorder. The available drugs include three acetylcholinesterase inhibitors (donepezil, rivastigmine and galantamine) and one N-methyl-D-aspartate (NMDA) receptor inhibitor (memantine). These two classes of medication target, respectively, cholinergic and glutaminergic neurotransmitter derangements commonly associated with AD. However, neither class of medication is thought to significantly alter the causal pathways in AD or prolong the lives of patients with the disease. Given the rapidly ageing demographic profiles in most areas of the world, new therapeutic interventions for AD are urgently needed that can slow or perhaps even prevent disease progression; ideally these treatments would restore normal brain function.

In recent years, a number of drug candidates targeting amyloid-ß (Aß) peptide have advanced into randomized controlled clinical trials. These include tarenflurbil (Myriad Genetics, Salt Lake City, UT, USA), semagacestat (Eli Lilly and Company, Indianapolis, IN, USA), tramiprosate (Neurochem Inc., Laval, Canada), ELND006 and AN1792 (Elan Corporation, Dublin, Ireland) and ponezumab (Pfizer, New York, NY, USA). However, most have failed because of safety issues or lack of efficacy 1. Building upon the lessons learned from these failures, other drug development programmes are being carried out with the aim of finding safer and more effective treatments for AD.

Understanding of the pathogenesis of AD has increased greatly since the early 1990s, giving rise to optimism that better treatments can now be developed. AD is currently perceived as a protein aggregation disorder. The Aß peptide is central to the pathogenesis 2, initiating and driving a cascade that leads to the dysfunction of neurons and, finally, to dementia. Several mutations in rare familial forms of the disease result in early-onset AD, either by increasing Aß production or by elevating the Aß42/40 ratio. Increased Aß levels accelerate aggregation of the peptide. Recently, a rare polymorphism for the Aß precursor protein (AßPP) has been identified that appears to decrease synthesis of Aß by approximately 40% and reduces the risk of AD 3. The production, aggregation and clearance of Aß are thus all attractive and feasible targets for drug development. Enzymes such as ß- or γ-secretase, which regulate the processing of AßPP and Aß production, can be inhibited by small molecules. However, it has proven difficult to make γ-secretase inhibitors that are specific for the target substrates and at the same time are nontoxic, and β-secretase inhibitors remain early in development 4–6.

Furthermore, the most prevalent late-onset sporadic form of AD is not clearly associated with Aß overproduction and may be more closely related to decreased Aß clearance 7. In this context, the value of using secretase inhibitors that lower Aß production without increasing clearance to treat AD is uncertain. Intervention designed to improve the clearance of Aß and/or prevent its accumulation are therefore being tested.

## Soluble forms of Aβ: a more appropriate target?

Due to their amphipathic nature, Aβ monomers tend to spontaneously aggregate and form larger soluble molecular species (oligomers/protofibrils). Further assembly leads to the formation of insoluble fibrils that eventually precipitate in the brain. Early-onset mutations such as the Arctic (*AßPP* E693G) mutation 8,9 result in Aß peptides with an increased propensity to form Aß protofibrils (large soluble Aß oligomers) without increasing total Aβ levels. Even in the absence of genetic mutations, the concentrations of Aβ oligomers are considerably higher in the brains of patients with AD than in those of similarly aged healthy individuals 10.

Of interest, the levels and distribution of soluble forms of Aβ better correlate with disease severity than those of insoluble fibrils 11–13. Amongst the prefibrillar intermediate Aβ species, several oligomeric forms of various molecular sizes have been identified 14. Oligomeric Aβ has been shown to elicit adverse biological effects both *in vitro* and *in vivo* 15, suggesting that it plays a central role in AD pathogenesis. Furthermore, Aβ oligomers and protofibrils have been shown to be toxic to neurons and synapses and to inhibit mechanisms associated with memory. For these and other reasons, the soluble and highly toxic forms of Aβ such as oligomers and protofibrils may be more directly linked to cellular pathology and are therefore appropriate targets for treatments; however, the equilibria between monomeric Aβ, oligomers or protofibrils and insoluble Aβ fibrils remain poorly understood.

## Anti-Aß immunotherapy

With only symptomatic treatment available, efforts to develop novel therapeutics aimed at lowering the amount of Aß peptides in the affected brain have intensified. In particular, immunotherapy against Aß has attracted considerable interest in recent years as it offers the possibility to generate molecules targeting highly specific moieties. Recent advances in protein engineering and the production of recombinant proteins make it feasible to produce tailor-made antibodies at a reasonable cost for therapeutic use.

Active immunization and passive immunization have been the most widely studied approaches to immunotherapy of AD over the past decade. A third approach known as immune modulation is also being explored but is in an earlier stage of investigation. Active immunization involves administration of a vaccine containing antigens or other stimuli designed to induce an immune response that generates antibodies in the recipient. In passive immunization, antibodies are delivered from a source other than the patient's own immune system, such as humanized murine monoclonal antibodies or donor-derived human polyclonal antibodies. Immune modulation involves the use of cytokines or other molecules designed to alter the functions of the immune system. Although immune modulation may secondarily affect processing of Aß, the focus of most research over the past decade has been active and passive immunotherapy strategies designed to address more directly the toxicity and clearance of Aß.

Active and passive approaches to immunotherapy both have advantages and disadvantages. An advantage of active immunization is the capacity for a small number of vaccinations to generate a prolonged antibody response. Antibodies raised in response to vaccination are generally polyclonal, so antibodies with multiple specificities against Aß can be produced. These antibodies may improve in specificity and/or affinity over time as a result of clonal maturation. A potential disadvantage of active vaccination is the variability in the antibody response across patients. This may be especially problematic in the context of late-onset AD owing to age-related reductions in the immune competency of elderly patients. The senescent immune system is less likely to generate therapeutically adequate titres of antibodies in response to vaccination and more likely to develop autoimmune side effects. Additionally, adverse effects can occur after active vaccination, which may depend on the binding epitopes of the polyclonal antibodies produced; these adverse effects can be persistent, even lifelong. For these reasons as well as others, the development of active immunization for AD has proceeded more slowly than that of passive immunization.

The potential advantages of passive immunotherapy include the reproducible delivery of a known amount of therapeutic antibodies to the patient and rapid clearance of those antibodies if side effects develop. A disadvantage is the requirement for repeated infusions of antibodies over time. The benefits of either active or passive immunization may be inhibited by anti-idiotype antibodies (neutralizing antibodies) that block the desired therapeutic effects. Ongoing AD programmes include agents for passive (Table1) as well as active (Table2) immunotherapy in various stages of development after Phase I.

**Table 1:** Passive immunotherapy studies for Alzheimer's disease (AD) beyond clinical Phase I as of January 2013

Company	Drug (type of antibody)	Aβ epitope	Clinical stage	Comment
Elan/Wyeth/Pfizer	Bapineuzumab (humanized monoclonal)	N-terminal	Two Phase III studies completed	Terminated for lack of efficacy
Eli Lilly	Solanezumab (humanized monoclonal)	Mid-domain	Two Phase III studies completed	Negative primary outcomes, positive effects in mild AD; another Phase III study planned
ADCS/Baxter	Gammagard IVIG (human polyclonal)	Aβ aggregate conformational neoepitopes	Phase III	Negative primary outcomes, positive effects in moderate AD and APOE e4 carriers
Roche/Morphosys	Gantenerumab (human monoclonal)	N-terminal and mid-domain+	Phase III	Conformational antibody
Pfizer	Ponezumab (humanized monoclonal)	C-terminal	Phase II	Terminated IgG2a
Genentech/Roche	Crenezumab (humanized monoclonal)	Soluble Aβ and plaques	Phase II	IgG4 subclass, with reduced effector function
Eisai/BioArctic	BAN2401 (humanized monoclonal)	Aβ protofibrils	Phase IIb	Phase II study in MCI/early AD

ADCS, Alzheimer's Disease Cooperative Study; IVIG, intravenous immunoglobulin; MCI, mild cognitive impairment.

**Table 2:** Active immunotherapies in Alzheimer's disease, in clinical Phase II, as of January 2013

Company	Drug	Aβ epitope	Clinical stage
Alzheimer immunotherapy	ACC-001	N-terminal, Aβ1-6	Phase II
Novartis/Cytos	CAD106	N-terminal, Aβ1-6	Phase II
GSK/Affiris	AFFITOPE AD02	N-terminal, Aβ1-6	Phase II

## Mechanisms of anti-Aß immunotherapy

One of the attractive features of anti-Aß immunotherapy is the multiplicity of molecules and the variety of mechanisms that can be targeted for therapeutic purposes. The versatility of Aß immunotherapy is a reflection of the inherent diversity of the human immune system, which has evolved the capacity to respond to an immense number of disease states. Antibodies can be directed against AβPP, the monomeric Aß molecule and many soluble and insoluble Aβ aggregation intermediates. Even non-Aß species, such as Aß carrier proteins and transport channels, are potential targets. This versatility is extremely important in the light of the uncertainty about which forms of Aß are pathogenic in AD and about the possible role that Aß monomers may play in normal human physiology 16.

There are no known active transport systems for antibodies of immunoglobulin (Ig)G isotype into the central nervous system (CNS) in humans. The neonatal Fc receptor acts as a pump to remove antibodies that are generated intrathecally or find their way into the CNS by passive diffusion, although generally the mechanisms for clearance of antibodies from the CNS are not well understood. As a consequence, only a small fraction (approximately 0.1%) of the antibodies introduced into the peripheral circulation can be detected in the brain or cerebrospinal fluid (CSF). The precise conduits for transport of antibodies into the CNS have not been fully identified but include the lymphatic system, perivascular spaces and areas within the CNS in which the blood–brain barrier (BBB) is leaky.

**Figure 1:**
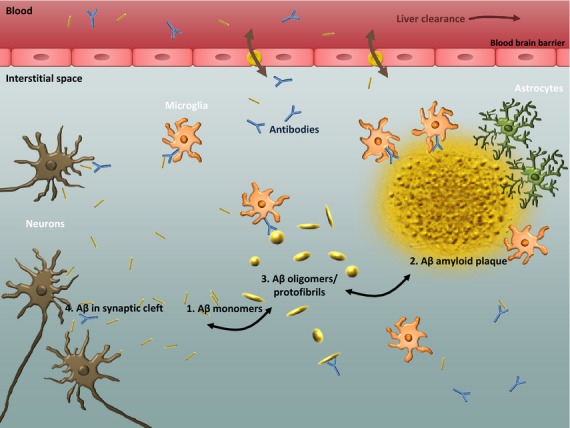
Possible targets for immunotherapy: 1. binding to soluble forms of Aß and increase clearance/shift equilibria. 2. Binding to the deposited amyloid plaque and promote plaque removal though microglial activation. 3. Binding to oligomers/protofibrils of Aß and clearing these species. 4. Enter into the synaptic clefts between neurons, and interfere with cell-to-cell transmission of Aß and its aggregates.

There are several loci at which immunotherapy targeting Aß can exert its effects (Fig.1). Even without reaching the brain, the presence of large amounts of anti-amyloid antibodies in peripheral circulation can create a driving force for movement of Aß out of the CNS. Antibodies in circulation contribute to the equilibrium between Aß in the blood and CNS compartments. If antibody levels are raised, passive diffusion down a concentration gradient can help to clear monomeric Aß from the brain. This mechanism, often referred to as the ‘peripheral sink hypothesis’, has been demonstrated in animal models 17, but its applicability to AD therapeutics is unclear.

Antibodies can alter Aß clearance by interacting with the transport system that moves Aß into and out of the CNS compartment 18. Influx into the CNS occurs via the receptor for advanced glycation endproducts (RAGE), and low-density lipoprotein receptor (LPR) is thought to be involved in its efflux. Other channels, carriers and receptors are also involved. Antibodies that block RAGE could in theory stimulate reduction in CSF Aß levels by preventing the movement of Aß from the blood into the brain 19.

The relatively small amounts of Ig that reach the brain after peripheral administration can exert a variety of effects on Aß in the CNS. For antibodies binding to Aβ, the type of effect is likely to be influenced by the binding epitope of the antibody, for example, antibodies that bind to soluble forms of Aβ may increase clearance and shift equilibria, whereas those that bind to the deposited amyloid plaque may require microglial activation to achieve plaque reduction 20. Other antibodies may disrupt or promote Aß aggregation or interfere with Aß binding to other molecules and thereby reduce toxicity. Antibodies might also bind to receptors on immune effectors and act as signals to generate or retard inflammation. Finally, antibodies might even be internalized in cells or enter into the synaptic clefts between neurons, with the potential to interfere with cell-to-cell transmission of Aß and its aggregates 21.

Although immunotherapy has not been widely considered as a method of reducing Aß production, recent preclinical studies have demonstrated the possibility of doing so through the use of antibodies that interfere with ß-secretase activity. In particular, antibodies that bind near the N-terminus of the Aß molecule or near the β-secretase cleavage site on the AßPP molecule have been shown to block β-secretase binding and thereby reduce Aß production 3. Because some of the cleavage of AßPP occurs at the neuronal membrane, it is not necessary for antibodies to become internalized within neurons to exert this effect. However, certain antibodies against AßPP may be internalized by neurons and act to reduce intraneuronal levels 22. Whether such approaches will prove viable for AD therapeutics remains to be determined.

## Early efforts: the AN-1792 vaccine trial

In a striking discovery that jump-started the field of AD immunotherapy, researchers at Elan demonstrated that active vaccination with fibrillar Aß generated anti-Aß antibodies that could alter fundamental components of AD neuropathology in novel ways 23. Vaccination with Aß fibrils plus an immune-stimulating adjuvant led to both the prevention of formation of new amyloid deposits and clearance of existing ones in the brains of transgenic mice over-expressing AßPP. These dramatic therapeutic effects in the absence of side effects in animals led to the rapid initiation by Elan of human clinical trials of the vaccine known as AN1792. Although initially thought to be promising, clinical trials of AN1792 were halted in Phase II because of the development of a T-cell-mediated meningoencephalitis in approximately 6% of the vaccinated patients with AD. This was subsequently found to be due to AN1792 stimulating a pro-inflammatory T helper (Th) 1-type immune response. This finding has led subsequent vaccine developers to attempt to generate immune responses which are purely humoral or involve Th2 stimulation rather than Th1. Another setback in this study was that only approximately 20% of those vaccinated raised antibody titres above the preset therapeutic cut-off level 24. This may be attributed to the elderly having reduced responses to vaccines of many kinds as a result of immune senescence.

In a postmortem examination of the brains from several study participants who received AN1792, fewer amyloid plaques and cerebral vascular deposits (amyloid angiopathy) were demonstrated than would be expected in an individual with long-standing AD. In some cases, almost no insoluble amyloid deposits from the brain were found 25. In addition, concentrations of total tau protein, a biomarker associated with neuronal loss, were slightly reduced in the CSF 24. Despite these apparently favourable biological responses, clinical outcomes were no better than those of the placebo-treated control subjects. Surprisingly, rates of brain atrophy as determined by volumetric magnetic resonance imaging (MRI) were greater in vaccinated than in control patients 26,27, possibly due to removal of Aß.

The unexpected side effects, lack of clinical efficacy and weak antibody response led many researchers to focus on passive immunotherapy in the aftermath of the AN1792 trial. However, there may have been some clinical benefits of AN1792 as a reduced functional decline was observed compared with placebo-treated patients 27. Several antibodies with affinity for various linear domains in the primary sequence of the Aß molecule (N-terminus, central region, C-terminus) entered into clinical trials in the years that followed 28.

## Passive immunotherapy

An approach for the treatment of AD is to reduce the build-up of amyloid plaque using monoclonal antibodies. Recently, clinical trial results from two late-stage monoclonal antibodies, bapineuzumab (Janssen Alzheimer Immunotherapy and Pfizer) and solanezumab (Eli Lilly and Co) for the treatment of mild-to-moderate AD were released.

### Targeting fibrillar forms of Aß: bapineuzumab

Bapineuzumab, a humanized monoclonal antibody, was designed to bind and remove the Aß peptide deposits that accumulate in the brain. Bapineuzumab binds to the N-terminus region of Aß, a region that typically remains exposed when Aß fibrils are formed, and binds to fibrillized Aß in plaques more strongly than to soluble Aß. Bapineuzumab was administered intravenously to AD patients with mild-to-moderate disease in two large, Phase III, multicentre, randomized, double-blind, placebo-controlled, parallel-group, efficacy and safety studies. The two trials differed in inclusion criteria and dosing. In the first trial, APOE ε4 carriers were enrolled and a reduced dosing regimen was employed with the aim of mitigating the side effects (see below) seen most frequently amongst ε4 carriers in the Phase II bapineuzumab trial. In the second Phase 3 trial, only non-ε4 carriers were enrolled. After 18 months, the primary outcome measures were no different in bapineuzumab-treated patients than in those who received placebo. Secondary outcomes were likewise largely negative. The study did provide evidence of target engagement and a small but statistically significant reduction in CSF levels of phospho-tau.

Bapineuzumab treatment was associated with a significant number of cases of vasogenic oedema, now termed amyloid-related imaging abnormalities with parenchymal oedema (ARIA-E), as well as intracerebral microhaemorrhages (ARIA-H). ARIA-E is characterized by areas of increased signal on some MRI pulse sequences thought to be due to excess water, presumably as a result of leakage at the BBB. These cases were observed despite attempts to reduce their occurrence by administering lower doses to apolipoprotein E ε4 carriers 29. In the light of the negative clinical outcomes and adverse event profile, the development of bapineuzumab has been terminated.

### Targeting soluble monomeric Aß: solanezumab

Solanezumab is a humanized monoclonal antibody that recognizes the middle region of Aß and binds soluble monomeric forms of the peptide. Solanezumab is administered intravenously and, as noted above, about 0.1% crosses the BBB into the CSF. The differences in binding epitopes for solanezumab and bapineuzumab result in different properties; solanezumab recognizes a mid-domain epitope and binds selectively to soluble Aß, whereas bapineuzumab binds to the N-terminal region of Aβ and also binds to deposited amyloid plaques. These different properties may account for the lower rate of ARIA-E reported for solanezumab compared with bapineuzumab 30,31.

Lilly has completed two multicentre, randomized, double-blind, placebo-controlled Phase III clinical trials of solanezumab as a potential treatment to slow the progression of mild-to-moderate AD. The trials, EXPEDITION and EXPEDITION-2, were identical in design; each included a randomized double-blind treatment period of 18 months during which patients were given intravenous solanezumab (400 mg) or placebo every 4 weeks. Together, the studies enrolled 2052 patients from 16 countries. Neither study met the prespecified primary outcomes; however, for patients with mild AD (MMSE = 20–26), a reduction in rate of cognitive decline was demonstrated in the EXPEDITION study using the ADAS-Cog_14_. This finding in patients with mild disease did not reach statistical significance for EXPEDITION-2, although pooled data from both studies for this group of patients showed a 34% reduction in cognitive decline. An open-label extension (EXPEDITION-EXT) study for patients who completed the EXPEDITION trials is ongoing, and an additional study in patients with mild AD is being planned. Solanezumab is also being tested for prevention of AD as part of the Dominantly Inherited Alzheimer's Network (DIAN) initiative which will involve administration to patients carrying autosomal dominant AD-causing mutations.

### Targeting soluble aggregates: intravenous immunoglobulin

Intravenous immunoglobulin (IVIG) is a polyclonal antibody preparation derived from the blood plasma of thousands of healthy donors. It has been used clinically as a replacement therapy for various immunodeficiency syndromes as well as for treatment of certain forms of cancer, haematological diseases and autoimmune disorders. IVIG contains the majority of IgG antibodies in the human repertoire, approximately 0.5% of which bind to Aß. Naturally occurring human antibodies in IVIG exhibit very little binding to monomeric Aß and instead recognize conformational neoepitopes found in Aß aggregates such as oligomers and fibrils 32. IVIG also exerts potent immune modulatory properties that may be linked to Aß binding or represent an independent mechanism of action.

Open-label IVIG treatment for 6 months provided symptomatic benefits in two Phase II trials 33,34. In a Phase II futility study involving 24 patients with mild-to-moderate AD, patients treated with Gammagard IVIG (Baxter) for 6 months did better on measures of cognition and global outcomes than those given placebo. The US National Institute on Aging (NIA) and Baxter funded a multicentre, randomized, double-blind, placebo-controlled Phase III trial of IVIG for mild-to-moderate AD, the Gammaglobulin Alzheimer Partnership (GAP) study, that was recently completed in the USA and Canada. A total of 390 patients with AD enrolled in the GAP study, the aim of which was to evaluate the safety and efficacy of two doses of Gammagard IVIG compared with placebo over 18 months 35. After the GAP study had successfully passed the important step of an interim futility analysis in early 2012, Baxter announced plans to initiate a confirmatory Phase III trial in the USA, Europe and Asia. On 7 May 2013, in a press release, Baxter disclosed that the primary end-points for the GAP study were unfortunately not met and that ongoing studies of Gammagard IVIG in patients with mild-to-moderate AD would be discontinued.

### Targeting Aß with a conformational antibody: gantenerumab

Gantenerumab (Roche) is a fully human anti-Aß antibody that has a high capacity to specifically bind to cerebral amyloid plaques. The antibody has two binding sites in Aß, one N-terminal, but it is also binding a region in the middle portion of the peptide 36. Results from Phase I clinical trials demonstrated that gantenerumab treatment resulted in a dose-dependent reduction in brain Aß in the Phase I trial, based on positron emission tomography (PET) imaging using a ligand that binds to deposited fibrillar plaques. Gantenerumab may act through phagocytosis via brain microglial cells. Amyloid load decreased in patients receiving active drug. ARIA-E was seen in some of the patients on high doses 37.

Roche is currently recruiting 360 patients for a multicentre (in 15 countries), randomized, double-blind, placebo-controlled, 2-year Phase II study. The aim is to investigate the efficacy, in terms of cognition and function, and safety of subcutaneous gantenerumab in patients in the early or prodromal stage of AD. Gantenerumab is also being tested for prevention of AD as part of the DIAN initiative (see above).

### Targeting Aß with reduced effector functions: crenezumab

Crenezumab is a fully humanized IgG4 monoclonal antibody against Aß that binds both monomeric and oligomeric forms, inhibits aggregation and promotes disaggregation. The IgG4 subclass leads to reduced effector functioning, that is, reduced Fc-mediated phagocytosis, and possibly less inflammatory reactions. AC Immune licensed its antibody crenezumab to Genentech in 2006. Crenezumab is currently being investigated in a Phase II, randomized, double-blind, placebo-controlled study in mild-to-moderate AD. This ongoing trial will enrol more than 370 patients in multiple centres worldwide, and the primary outcome measures are cognitive and global function. The antibody is also going to be tested for AD prevention in a large Colombian cohort with a presenilin-1 mutation, in Alzheimer's Prevention Initiative (API). Genentech has responsibility for clinical development, manufacturing and commercialization of the antibody.

### Targeting Aß protofibrils: BAN2401

In recent years, there has been increased interest in soluble oligomeric assemblies of Aß, rather than senile plaques, as a target for treatment of AD. In the late 1990s, the Arctic mutation was found in a family from northern Sweden. Studies of this mutation led to the realization that its pathogenic effect was to generate large toxic soluble Aß oligomers, that is, protofibrils 8. An attempt was made to target Aß protofibrils with immunotherapy. The development of a conformation-dependent antibody with the ability to recognize a unique structure in the Aß protofibril was undertaken 38,39. Most other research groups have developed therapeutic Aß antibodies that bind to a linear epitope in Aß 28.

To investigate the phenotype of an antibody with regard to its binding properties, it is important to perform the experiment in solution. mAb158 was selected using an inhibition enzyme-linked immunosorbent assay format where the antibody–antigen interactions take place in solution and at low concentrations 38,40. Treatment with mAb158 decreased levels of soluble Aβ protofibrils in both young (4 months) and elderly (14 month) tg-ArcSwe mice, whilst Aβ plaque pathology was reduced only when the treatment was initiated before plaque onset 41. Despite substantial amounts of insoluble plaque, it is feasible to selectively clear soluble Aβ aggregates from the brain, although insoluble Aβ constitutes an overwhelming majority of the total Aβ content.

BioArctic Neuroscience has produced BAN2401 (humanized version of mAb158) and further developed the immunotherapeutic strategy. In 2007, BioArctic entered into a licence agreement with Eisai of Japan, with the aim of bringing BAN2401 to the world market. A Phase I/IIa clinical safety study was initiated in 2010 in 80 patients with AD (60 treated with BAN2401 and 20 with placebo). Both single-ascending and multiple-ascending doses were administered with a highest dose of 15 mg kg^−1^. No serious adverse events were observed, and Phase IIb was started in January 2013 in patients with mild cognitive impairment (MCI) and early AD.

## Active immunotherapy

At least three agents for active immunotherapy of AD have reached Phase II of clinical development.

### Acc-001

Janssen Immunotherapy is developing a second-generation Aβ vaccine, ACC-001. The vaccine is composed of Aβ1-6 attached to a carrier protein, using the saponin adjuvant QS-21. ACC-001 induced an antibody response without intolerable side effects in a Phase I study and is currently being evaluated in a Phase II clinical trial.

### Cad106

In 2001, Cytos Biotechnology announced the development of CAD106 in collaboration with Novartis. CAD106 is another Aβ vaccine in Phase II clinical trials involving patients with mild AD. It consists of two components, the Immunodrug carrier Qb coupled with a fragment of the Aβ protein. In animal studies, it has been shown that treatment with CAD106 can block the formation of Aβ plaques in the brain. The first Phase II trial was a randomized, double-blind, placebo-controlled study to evaluate the safety and tolerability of CAD106 when administered as repeated subcutaneous injections in subjects with mild AD 42. A favourable safety profile was seen. The second Phase II study of CAD106 was designed as a nonrandomized, open-label, double-blind, placebo-controlled, single-group trial to determine the safety and tolerability of CAD106 in patients with AD.

### AFFITOPE AD02

AFFITOPE AD02 is an immunogen consisting of the amino-terminal B-cell epitope of Aβ that was designed to avoid the T-cell activation issues that led to meningoencephalitis with the AN1792 vaccine. This vaccine is under development by Affiris in collaboration with GlaxoSmithKline and has reached Phase II development. In preclinical studies in murine models of AD, AFFITOPE AD02 reduced total plaque area as well as astrocytic and microglial activity relating to plaques. Results of Phase I testing in 24 patients with AD were announced in 2011, and AD02 met the primary safety and tolerability end-points. A Phase II study of AD02 involving 400 patients with AD is being conducted in multiple centres in Europe.

## Current challenges and future directions

After more than a decade of research, anti-amyloid immunotherapy remains one of the most promising emerging strategies for developing disease-modifying treatments for AD. However, active and passive immunotherapy agents for AD have yet to be approved. Several key issues remain to be addressed with regard to their further development.

### What are the right disease targets?

The initial impetus for testing immunotherapy in AD was the discovery in murine models that amyloid-containing brain plaques could be removed and even prevented by anti-Aβ antibodies. Amyloid plaque burden appeared to be reduced based on autopsy studies of patients with AD treated with AN1792 as well as on PET imaging of patients treated with bapineuzumab. However, neither of these agents brought about clinical improvements and both were associated with side effects. The time of appearance and the distribution of plaques in the brain do not correspond to the onset of clinical symptoms of AD; plaque deposition may begin at least 10 years prior to the onset of cognitive loss 43,44. As such, plaque removal may not be a desirable target for treatment of symptomatic AD, although it could still prove to be a viable target for prevention. Soluble aggregates of Aβ such as oligomers and protofibrils are highly toxic to the brain and are logical targets for anti-Aβ immunotherapy. However, these soluble Aβ aggregates tend to be short-lived, low-abundance molecules that are more difficult to characterize and target than the relatively stable monomers and fibrils. Innumerable oligomeric species exist in a complex relationship along the pathways of Aβ assembly. A better understanding of the nature and mechanisms of toxicity of the soluble Aβ aggregate intermediates that contribute to the pathogenesis of AD could help to advance the development of more potent anti-amyloid immunotherapies.

### Is active or passive immunotherapy the best approach?

Both active and passive anti-Aβ immunotherapies have shown potential benefits as well as adverse effects in AD clinical studies. In the light of the decreased response of the immune system of elderly patients to vaccinations, active immunotherapy may be best implemented in younger, at-risk individuals as part of prevention strategies or in patients with empiric evidence of the capacity for vaccine responsiveness. Passive immunotherapy may be advantageous as a route to developing treatments for AD owing to its ability to deliver known quantities of well-characterized antibodies against very specific epitopes. Whilst this approach may lower risks of irreversible autoimmune complications, there are high costs and practical limitations involved in infusing or injecting antibodies at regular intervals for what may need to be lifelong treatment. Nevertheless, at a time when AD prevalence is increasing rapidly and no disease-modifying therapy has been confirmed or approved for use, it can be argued that all possibilities for anti-Aβ immunotherapy need to be further explored.

### Who are the most appropriate subjects for immunotherapy studies?

After a decade of clinical trials with anti-Aβ agents, not a single new treatment for AD has been approved. The concept of testing candidate AD therapies in patients already suffering from mild-to-moderate dementia has come into question. Because amyloid deposition in the form of diffuse plaques begins as long as two decades prior to the onset of dementia symptoms, several pharmaceutical companies are turning their attention to prevention trials in asymptomatic, at-risk individuals as a means of targeting the disease at stages in which the predominant pathology is Aβ-related rather than due to other downstream elements of the amyloid cascade. Based on a similar rationale, symptomatic patients with mild cognitive impairment or very early AD are being enrolled in clinical trials. It remains to be determined whether focusing on prodromal and very early stages will increase the likelihood of success of immunotherapy for AD. Results from the solanezumab trials showing a probable slowing of disease progression in patients with mild but not moderate disease provide support for the concept that early treatment is important for amyloid-based therapies.

### Can new study designs accelerate discovery?

Alzheimer's disease clinical trial methodology was developed during the 1980s and 1990s for drugs that were quite different in mechanism and effect from anti-Aβ immunotherapy interventions. During the first decade of the 21st century, trial design has evolved in several ways to incorporate new understanding of the disease process and the benefits of new technologies for screening and assessing patients with AD. Most recently, new designs for AD prevention trials have been introduced that rely more on biomarker and imaging measures to assess target engagement and less on clinical outcomes. Whilst there is unquestionably a major need for AD prevention initiatives, abandoning attempts to develop new treatments for those already suffering from dementia could have a disastrous effect on prevalence in the next decades as large segments of the population reach the age of risk for AD. As such, improving the design of studies involving symptomatic patients with AD is an important goal.

One strategy for improving immunotherapy studies is the use of newly approved PET amyloid imaging technology as a means of identifying individuals who have measurable brain amyloid burden, and thus improving diagnostic accuracy, which is being done in the Alzheimer's Disease Cooperative Study (ADCS) A4 trial. This is particularly important because results from the bapineuzumab and solanezumab trials suggest that as many as 30% of enrolled subjects may have received non-AD diagnoses.

Attrition is another important issue in the validity of AD immunotherapy trials. Past trials of symptomatic treatments lasted 3–6 months and typically experienced attrition rates of 10–15%. However, to monitor for potential disease-modifying effects, immunotherapy trials that are 18–24 months in duration have attrition rates as high as 30–40%. Shorter duration trials that achieve similar goals without high attrition may become possible with increased use of biomarker and imaging outcomes; however, regulatory agencies have not yet approved drugs based on biomarker findings rather than clinical measures, but this might change in future. Biomarkers as primary outcomes may have greater utility in Phase II studies, given the current regulatory environment.

### What is the role of biomarkers and brain imaging in testing immunotherapy?

Immunotherapy trials have used biomarkers in blood and CSF as means of demonstrating target engagement, but there have been few successes in correlating biomarker and clinical outcomes. In Phase I and II AD trials in general and immunotherapy trials in particular, biomarkers may be very useful to demonstrate target engagement and dose–response relationships. However, one problem that hampers interpretation of biomarker studies is a lack of consensus about the direction and magnitude of change in a biomarker that is necessary to predict a clinical effect. For example, depending on the agent under study and the assay methods employed, Aβ levels in plasma and CSF may go up, down or stay the same after treatment. A rigorous understanding of mechanisms of action and shifts in Aβ equilibria are needed to interpret meaningfully biomarker signals in immunotherapy and other AD therapeutic trials.

In the meantime, imaging and CSF biomarkers can play an important role in improving the accuracy of diagnosis in clinical trial participants and permit enrolment of subjects in prodromal stages prior to the development of frank dementia symptoms. There are currently no accepted surrogate biomarkers for AD progression. Nevertheless, correlations between clinical outcomes and imaging and fluid biomarkers are desirable for better characterizing disease-modifying effects of immunotherapy interventions.

## Conclusions

The findings from genetic, longitudinal CSF biomarker 45,46 and recent cohort studies using imaging and biomarker methods 47 all suggest that Aβ accumulation is one of the earliest events in the pathogenesis of AD. However, the results of clinicopathological studies in which the frequency and location of senile plaques have been compared with cognitive dysfunctions in postmortem AD brain samples indicate that senile plaques are not the main cause of dementia 48. Soluble Aβ species may be the most direct cause of AD pathogenesis. Aβ aggregation and fibril formation are part of a complex multistep process, and it has been difficult to model the individual steps in this process *in vitro* and *in vivo*. Several immunotherapeutic strategies involving active and passive immunization are now being tested in clinical trials. Whilst it is difficult to predict which one of these will be most successful, there is considerable cause for optimism that immunotherapy will lead to better treatment and prevention of AD in future.

## Conflict of interest statement

Lars Lannfelt is co-founder of BioArctic Neuroscience AB and Chairman of the Board. Norman Relkin is Project Leader for the NIA-Baxter GAP study of IVIG for AD and a consultant to Eisai and Kirin on AD immunotherapy studies. Eric Siemers is an employee and shareholder of Eli Lilly and Co.
